# A Behavioral Taxonomy of Loneliness in Humans and Rhesus Monkeys (*Macaca mulatta*)

**DOI:** 10.1371/journal.pone.0110307

**Published:** 2014-10-29

**Authors:** John P. Capitanio, Louise C. Hawkley, Steven W. Cole, John T. Cacioppo

**Affiliations:** 1 Department of Psychology & California National Primate Research Center, University of California Davis, Davis, California, United States of America; 2 National Opinion Research Center, University of Chicago, Chicago, Illinois, United States of America; 3 Department of Medicine, Division of Hematology-Oncology, University of California Los Angeles School of Medicine & the Norman Cousins Center, University of California Los Angeles, Los Angeles, California, United States of America; 4 Department of Psychology & Center for Cognitive and Social Neuroscience, University of Chicago, Chicago, Illinois, United States of America; University of Portsmouth, United Kingdom

## Abstract

Social relationships endow health and fitness benefits, but considerable variation exists in the extent to which individuals form and maintain salutary social relationships. The mental and physical health effects of social bonds are more strongly related to perceived isolation (loneliness) than to objective social network characteristics. We sought to develop an animal model to facilitate the experimental analysis of the development of, and the behavioral and biological consequences of, loneliness. In Study 1, using a population-based sample of older adults, we examined how loneliness was influenced both by social network size and by the extent to which individuals believed that their daily social interactions reflected their own choice. Results revealed three distinct clusters of individuals: (i) individuals with large networks who believed they had high choice were lowest in loneliness, (ii) individuals with small social networks who believed they had low choice were highest in loneliness, and (iii) the remaining two groups were intermediate and equivalent in loneliness. In Study 2, a similar three-group structure was identified in two separate samples of adult male rhesus monkeys (*Macaca mulatta*) living in large social groups: (i) those high in sociability who had complex social interaction with a broad range of social partners (putatively low in loneliness), (ii) those low in sociability who showed tentative interactions with certain classes of social partners (putatively high in loneliness), and (iii) those low in sociability who interacted overall at low levels with a broad range of social partners (putatively low or intermediate in loneliness). This taxonomy in monkeys was validated in subsequent experimental social probe studies. These results suggest that, in highly social nonhuman primate species, some animals may show a mismatch between social interest and social attainment that could serve as a useful animal model for experimental and mechanistic studies of loneliness.

## Introduction

For social species, having and maintaining social relationships with conspecifics is critical to individual survival and well-being. Among humans, the benefits of social connection include reduced morbidity and mortality [1], [2], better physiological function [3], and improved mental health [4], [5]. Similar results have been found for nonhuman primates [6], [7], with studies also documenting positive fitness consequences of close social bonds [8].

Considerable variation exists, however, in the extent to which individuals form and maintain salutary social relationships [9]. These variations have often been analyzed in terms of broad personality traits, such as introversion. However, introversion rarely emerges as a strong risk factor for individual outcomes such as well-being. Instead, the most toxic effects are often associated with *perceived* isolation (i.e., loneliness; [9]–[13]). Whereas introversion refers to the preference for low levels of social involvement [14], loneliness refers to the perception that one's social relationships are inadequate in light of their preferences for social involvement. One can feel lonely whether alone or in a crowd. Increased feelings of loneliness, whether experimentally induced or naturally occurring, cause people to feel not only unhappy but also unsafe, heightening their sensitivity to perceived social threats and attacks, and leading them to behave in a self-protective, overly reactive fashion [10]. Interestingly, many of these effects can be found in experimental studies of isolation in nonhuman social animals, as well [3].

Studies of twins indicate that loneliness is moderately heritable [15]–[18]. To address concerns that heritability estimates for loneliness from twin studies might not be generalized to the general population, Distel et al. [19] examined the genetic architecture of loneliness in an extended twin-family design. The presence of assortative (non-random) mating, genetic non-additivity, vertical cultural transmission, genotype-environment (GE) correlation and interaction were modeled. Results indicated the presence of positive assortative mating for loneliness – people who are similar in their trait loneliness tend to mate. Distel et al. [19] also confirmed that loneliness is moderately heritable, but interestingly found a significant contribution of non-additive genetic variation. Although situational determinants were identified, no evidence was found for vertical cultural transmission, which suggests that parents may also pass on genes for loneliness. Together, the architecture of loneliness suggests it may be a trait that was not neutral to selection in our evolutionary past.

Previous studies also indicate that there are environmental influences on the phenotypic variation in loneliness found in populations. For instance, freshmen who leave family and friends behind often feel increased social isolation when they arrive at college even though they are surrounded by large numbers of other young adults [20], [21]. Lower levels of loneliness are associated with marriage [22], [23], higher education [24], and higher income [24], [25], whereas higher levels of loneliness are associated with living alone [26], infrequent contact with friends and family [22], [27], [28], dissatisfaction with living circumstances [29], physical health symptoms and disabilities [30], chronic work and/or social stress [30], small social network [22], [28], lack of a spousal confidant [30], marital or family conflict [31], [32], poor quality social relationships [26], [28], [30], and divorce and widowhood [33]–[36].

Development of an animal model of loneliness would greatly facilitate analyses of the behavioral and biological effects of perceived social isolation [3], [37], and nonhuman primates, particularly monkeys of the Old World such as rhesus monkeys, are an excellent choice for model species [38]. Not only do Old World primates and humans share a recent common ancestor [39], which can facilitate finding common underlying biological mechanisms [40], but many species of Old World monkeys, like humans, are remarkably social, spending virtually their entire lives surrounded by multiple adult conspecifics (as well as animals of other age classes) of both sexes [41], [42]. Finally, like humans, nonhuman primates show naturally-occurring variation in levels of social interaction (i.e., Sociability) [43], and this variation is linked to differential behavioral outcomes measured years later in heterologous social contexts [44].

In this report, we first identify a classification scheme in humans reflecting level of social interaction and degree to which that level of interaction reflects the individuals' own choice. We next examine behavioral data from two samples of rhesus monkeys that map onto this classification, and conclude by describing social probe tests that behaviorally validate the classification of low-sociable monkeys into distinct groups that reflect differences in social interest, and may provide a nonhuman primate model of loneliness.

## Study 1—Social Network, Social Interaction, and Loneliness in Humans

Which behavioral markers in humans can be used to quantify variation in social interest that might co-vary with loneliness and can be adapted for use in an Old World monkey model? One objective measure might be level of social interaction, and in humans, this can be easily measured via self-report of social network size. This measure, however, is not a sufficient indicator of social interest; in humans, as in monkeys, a small social network may represent a social choice (as in introversion) rather than loneliness, and a large social network may represent a burden rather than salubrious social bonds. Therefore, in our human study, we determined the level of loneliness by dividing a population-based sample of older adults along the dimensions of large or small social network size *and* the extent to which individuals' levels of social interaction represented their own choices. We hypothesized that loneliness would be lowest in respondents with large social networks who believed their levels of social interaction reflected their own choice, and would be highest in respondents with small social networks who believed their levels of interaction were not their own choice. We additionally hypothesized that the differences in loneliness across these groups would be independent of individual differences in introversion or interpersonal anxiety.

### Methods

#### Participants

Participants were drawn from Year 5 of the Chicago Health, Aging, and Social Relations Study (CHASRS) - a longitudinal, population-based study of non-Hispanic White, African American, and non-Black Latino American persons born between 1935 and 1952. Of the 163 participants, 23 failed to provide data for one or more of the primary measures used in this study (i.e., loneliness, social choice, network size), resulting in a sample size of 140. See [9] for sampling design details. The protocol for the CHASRS study was reviewed and approved by the University of Chicago Institutional Research Board (Biological Sciences Division) and written consent was obtained from all participants.

#### Procedures

Participants completed standard psychological surveys, health and medication interviews, anthropometric measurements, and a cardiovascular protocol. Loneliness and social network size were measured in the first survey packet of the day, and participants reported on social choices in a daily diary completed at home at bedtime on each of three consecutive days.

#### Measures

Loneliness was assessed using the well-validated 20-item revised UCLA Loneliness Scale [21], [45]. Each item is rated on a scale of 1 (*never*) to 4 (*always*). After reverse scoring appropriate items, loneliness scores are calculated by summing across all items. Scores can range from 20 (low loneliness) to 80 (high loneliness).

Social network size was assessed by asking participants how many people (spouse, relatives, friends, neighbors) they interacted with at least once every two weeks. The mean network size among the 141 subjects who answered these questions was 11.5 (SD = 6.3).

In daily diaries, participants were asked how much time they spent that day: (a) alone, with no one around; (b) around others, but not communicating with them; and (c) with others, talking or listening to them. For each type of social situation, participants were asked to what extent the time was spent this way by choice (range  = 1, not at all my choice, to 5, completely my choice). Responses were highly correlated across the situations and days, so the nine responses were averaged (Cronbach's alpha  = 0.90). The mean degree of choice over social activity among the 151 subjects who answered these questions was 3.91 (SD = 0.94).

The validated 15-item Interaction Anxiousness Scale (IAS) of the Social Anxiousness Scale [46] served as our measure of interpersonal anxiety. Respondents are asked how characteristic each statement is of them on a scale of 1 (not at all characteristic of me) to 5 (extremely characteristic of me). IAS scores are summed after appropriate directional recoding, and can range from 15 (low anxiety) to 75 (high anxiety).

We used the 20-item Surgency (extraversion) subscale of the Big Five Personality Inventory [47] to assess introversion/extraversion. Subjects rated how accurately each of 10 positive and 10 negative trait words described themselves on a scale of 1 (extremely inaccurate) to 9 (extremely accurate). Extraversion scores were computed as the mean across the 20 appropriately coded items; lower scores indicate greater tendencies to introversion than extraversion.

#### Data analysis

Our goal was to investigate a possible behavioral taxonomy for nonhuman primate research. Although the data in Study 1 represent reliable individual differences along the measured continua, the reliable measurement of individual differences in nonhuman primate behaviors requires grosser measurement units. For this reason, we performed median splits to create a Social Choice (low, high) x Network Size (small, large) between-subjects factorial design to analyze the criterion measure (total score on the UCLA loneliness scale). (We note that treating Social Choice and Network Size as continuous variables in the analyses does not change the results.) To assess discriminant validity, we analyzed loneliness using analyses of covariance (ANCOVAs) to control for social anxiety and introversion. Data are presented as Supporting Information in file Data S1.

### Results and Discussion

**Figure 1:**
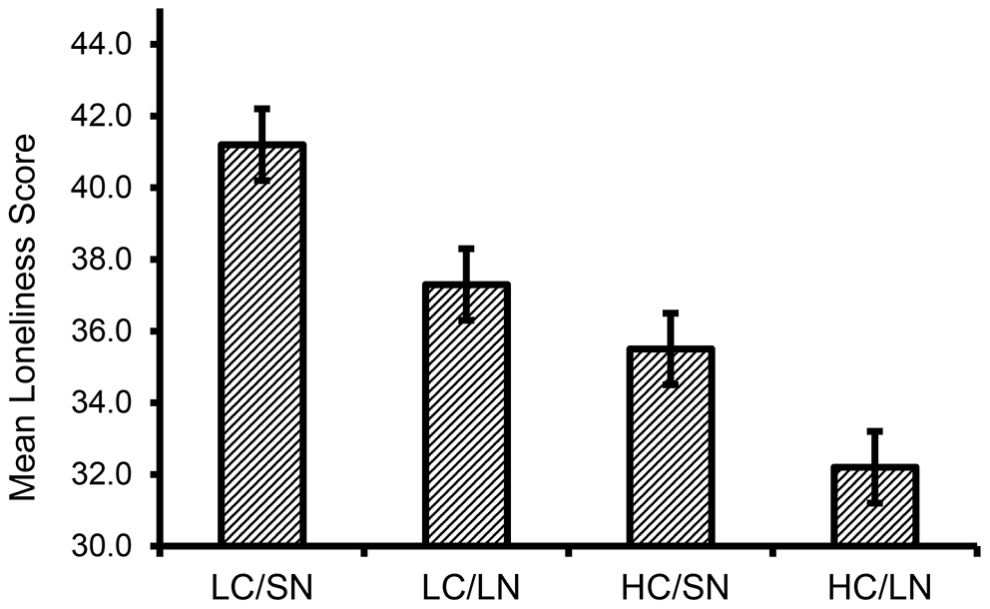
Group differences in loneliness among humans. Individuals with low social choice (LC) and small social networks (SN) have levels of loneliness that are significantly greater than individuals with high choice (HC) and large networks (LN). Individuals with low choice/large networks and those with high choice/small networks were not different from each other, but were significantly different from the other two groups.

A two-way analysis of variance showed main effects of choice (*F*(1,136)  = 19.82, *p*<.001, η_p_^2^ = 0.13) and network size (*F*(1,136)  = 9.02, *p*<.01, η_p_^2^ = 0.06), and a nonsignificant interaction (*p*>.8). Pairwise comparisons confirmed that loneliness was highest among those with small social networks and low choice over their levels of social interaction and was lowest among those with large social networks and high choice (Figure 1). Means for the small network/high choice and the large network/low choice groups fell between those of the other two groups, with both differing significantly from the other two groups (*p*'s<.05) but not from each other (*p*>.3).

The same group differences in loneliness were replicated in two-way ANCOVAs controlling for interpersonal anxiety (main effects for choice, *F*(1,134)  = 13.25, *p*<.001, and network size, *F*(1,134)  = 5.95, *p*<.05) or introversion (main effects for choice, *F*(1,133)  = 14.29, *p*<.001, and network size, *F*(1,133)  = 5.78, *p*<.05).

In sum, the multi-group human taxonomy was effectively reduced to three groups in which loneliness was highest among those with small networks who felt they had little choice over their levels of social interaction, lowest among those with large networks who felt they had more choice over their levels of interaction, and intermediate among those that possess either, but not both, high levels of social interaction and choice over those levels. Moreover, this tripartite grouping was independent of individual differences in introversion or interpersonal anxiety. In the remaining studies, we investigated the extent to which similar groupings were evident in monkeys (Study 2) and whether their behavior in response to social probes paralleled the differences that have been found among people who differ in their level of loneliness (Study 3).

Our human study showed that loneliness was highest among individuals that have low levels of social interaction, but who may be dissatisfied with those levels (low choice in determining those levels); in fact, individuals with comparably sized social networks, but who indicated that their amount of social interaction reflected their own choice (presumably reflecting satisfaction with their level of interaction), reported significantly less loneliness. Put another way, people who are lonely show a discrepancy between their social interest and social attainment. In a nonhuman species, this is likely to be most evident in animals that show low social attainment; we propose that animals that appear to be low in sociability can be differentiated into two groups by examination of the targets of social interaction (adult males, adult females, juveniles, infants), and the quality of the interaction with those targets. By “quality of interaction,” we distinguish between initiations that could be considered *tentative*, such as approaches, versus *complex*, such as grooming, which require a greater degree of tolerance by, and some coordination with, the target. Specifically, we propose that individuals that have low levels of social interaction (both tentative and more complex interaction) across all targets are likely to be relatively satisfied with their social situation, and might be considered “not lonely.” In contrast, low-sociable animals that show higher frequencies of tentative (but not complex) interaction, and whose tentative interaction is directed preferentially to targets that may be more likely to respond to overtures in an affiliative manner, might be the group that is most similar to the lonely humans, reflecting a discrepancy between their social interest and attainment.

Our studies were conducted with adult male rhesus monkeys born and reared in large, outdoor social groups. Each animal was observed for a fixed period of time to insure the human observers were equally familiar with all subjects. Occurrences of tentative (walkby, approach) and complex (groom, contact, proximity) affiliative behaviors were recorded along with the targets of behavior: adult males, adult females, juveniles, and/or infants. After the observations, animals were individually rated using a validated scale to ascertain individual levels of Sociability. Because our principal focus was on distinguishing between two groups of animals that had overall lower levels of affiliation, but that would be different based on occurrences of tentative social interaction, we identified low-Sociable animals, and subjected their approach and walkby data to a cluster analysis. We report below the results for two separate samples. Sample 1 was the sample in which we first identified the phenomenon of “lonely” monkeys; animals were observed and selected to participate in a study of personality (low- vs. high-Sociability) and simian immunodeficiency virus (SIV) infection [48]. Most data, however, were obtained prior to SIV inoculation (see below). Sample 2, our replication sample, was drawn for a study designed specifically to further explore “loneliness” in adult male monkeys; we present initial results from this ongoing study.

All studies conducted with rhesus monkeys were carried out in strict accordance with the recommendations in the Guide for the Care and Use of Laboratory Animals of the National Institutes of Health. The protocols were approved by the Institutional Animal Care and Use Committee of the University of California, Davis (Protocol numbers 9009, 15740). No monkeys were sacrificed for this project. The University of California, Davis, and the CNPRC are accredited by the Association for Assessment and Accreditation of Laboratory Animal Care.

## Study 2 – Classification of Rhesus Monkeys

### Methods

#### Subjects and housing

Subjects were n = 88 (Sample 1: S1) and n = 122 (Sample 2: S2) adult male rhesus monkeys, *Macaca mulatta,* that had been born and reared, and were currently living, in outdoor, half-acre enclosures at the California National Primate Research Center. Each enclosure contained up to 150 animals of all age/sex classes, in proportions approximating those of troops in the wild. All animals in these enclosures live outdoors in social groups all year-round. Cages contain a variety of structures used for climbing, socializing, and playing. Animals are fed chow twice daily, have water available ad lib, and receive a variety of enrichments regularly, including fresh vegetables at least twice per week, swimming pools during the summer, branches for climbing, etc. Members of Sample 1 were of intermediate and low rank and were a mean of 6.9 (range  = 4.9–9.8) years of age (because a subset of Sample 1 animals were to be selected for an infectious disease study, high-ranked animals were not permitted to be taken). Members of Sample 2 comprised animals of all ranks, and were a mean of 7.1 (range  = 4.2–18.7) years of age. (For comparison, we note that male rhesus monkeys in captivity reach sexual maturity at approximately 3.5 years of age, and are considered full-grown at about 8 years of age [49].) During the period of observations, all animals were healthy and weights ranged from 6.2 to 17.4 kg (mean  = 11.5 kg) for Sample 1, and 5.9 to 17.6 kg (mean  = 10.4 kg) for Sample 2.

#### Behavioral observations and personality assessment

Behavioral observations were conducted in the animals' familiar enclosures by trained observers who had demonstrated at least 85% agreement on scoring of behavior categories. Methods varied slightly, according to sample. For Sample 1, each animal was observed for twenty minutes per day (four 5-min sessions spread across a four-hour period each day) for 5 days, using focal animal sampling [50], and for Sample 2, animals were observed for two 10-min sessions per day for 8 days. For both samples, the occurrence of every behavior category observed was recorded within 15-sec intervals (total of 20 intervals per 5-min session for Sample 1 and 40 intervals per 10-min session for Sample 2), as well as the age/sex class (adult male, adult female, juvenile, infant) of interactants. After all behavioral observations were concluded for a given animal, the behavioral observers rated the animal on a 7-point Likert-type scale for each of 50 personality items [44]). Exploratory [51] and confirmatory [52] factor analysis of items yielded a “Sociability” factor comprising the items affiliative, warm, and solitary (reverse coded). Cronbach's alpha values for the z-scored scales were 0.92 (S1) and 0.93 (S2). “Low Sociability (LS)” and “High Sociability (HS)” were defined as Sociability factor z-scores less than -0.5 or greater than +0.5, respectively. The numbers of LS/HS animals were 31/29 (S1) and 30/38 (S2). For neither sample was there a relationship between classification and age (S1: p = .46; S2: p = .21). In Sample 1, mid-ranked animals were more likely to be HS than were low-ranked animals (*Chisq*(1)  = 4.35, *p*<.05, phi  = .269); in Sample 2, no effect of rank was found (*p* = .95).

Rating-based classifications showed expected correlates with objective indicators of social functioning, as has been shown in other samples [44]. ANOVA revealed that HS animals showed significantly higher frequencies of proximity with other animals (S1: *p*<.001; S2: *p*<.001), contact with others (S1: *p*<.001; S2: *p*<.01), and grooming initiated (S1: *p*<.001; S2: *p*<.05) compared to LS animals.

#### Data analysis

To assess the potential existence of naturally occurring clusters within the LS group we performed a two-group cluster analysis (K-means cluster analysis, SPSS Inc., version 22) on the behavioral measures of locomotion to within arm's reach that resulted in the animals remaining close for at least three seconds (approach) or for less than three seconds (walkby), respectively. These behaviors were each recorded as directed to four social targets (adult male, adult female, juvenile, infant); consequently the cluster analysis was performed with 8 variables. In the next two paragraphs, we report results of the ANOVAs for the cluster analysis, but caution that, because this procedure is specifically designed to identify clusters whose differences on these variables are maximal, these results are best interpreted descriptively and not as formal tests of significance of a null hypothesis. Where data did not meet the assumptions of ANOVA (for Studies 2 and 3), we used either Welch's test, log-transformed values, or nonparametric tests. Data are presented as Supporting Information in file Data S1.

### Results and Discussion

For Sample 1, the 31 LS animals were distributed in two groups, designated Manifestly Low Sociable (MLS; n = 5) and Truly Low Sociable (TLS; n = 26); group membership was unrelated to Sociability (*p* = .44), age (*p* = .97), or rank (*p* = .31). Inspection of the ANOVA table revealed that MLS animals had significantly higher frequencies for four measures, compared to TLS animals: MLS animals displayed approximately three times more walkbys to adult females (*p*<.01) and to juveniles (*p*<.001), and approximately twice as many approaches to adult females (*p*<.01) and juveniles (*p*<.001). Frequencies of walkby and approach to infants were also greater among MLS animals, though were not significant (*p*>.10); frequencies of walkby and approach to adult males were nearly identical for both groups of monkeys (see upper panel of Table 1).

**Table 1:** Mean (SD) frequencies for walkby and approach for MLS and TLS animals from Samples 1 and 2.

	Walkby				Approach			
	Male	Female	Juvenile	Infant	Male	Female	Juvenile	Infant
Sample 1								
MLS (n = 5)	4.6 (0.81)	10.8 (3.54)*	15.4 (3.02)*	1.0 (0.55)	5.4 (1.29)	13.4 (2.03)*	16.4 (2.50)*	2.4 (0.24)
TLS (n = 26)	4.8 (0.65)	3.5 (0.54)	5.7 (0.65)	0.3 (0.69)	5.4 (0.64)	6.3 (0.74)	7.6 (0.72)	1.6 (0.30)
Sample 2								
MLS (n = 5)	1.4 (0.25)	14.2 (1.91)*	7.2 (1.02)*	5.0 (1.18)*	0.4 (0.25)	15.4 (1.60)*	6.8 (2.18)	2.4 (0.51)*
TLS (n = 25)	0.8 (0.18)	2.3 (0.45)	3.1 (0.48)	0.5 (0.12)	2.2 (0.47)	4.7 (0.70)	4.7 (0.50)	0.6 (0.17)

Values shown are means and SD; significance tests were based on log-transformed values as needed.

*indicates significant differences (see text) between MLS and TLS animals.

The 30 LS animals from Sample 2 were clustered into MLS (n = 5) and TLS (n = 25), and results were very similar to those of Sample 1. Grouping was unrelated to Sociability (p = .26), age (*p* = .09), or rank (*p* = .76). Compared to TLS animals, MLS animals showed more walkbys to adult females (*p*<.001), juveniles (*p*<.01), and infants (*p*<.001), and more approaches to adult females (*p*<.001) and infants (*p*<.001). Frequencies for MLS animals in these categories were approximately 2–10 times greater than were frequencies for TLS animals. Unlike with Sample 1, TLS animals in Sample 2 showed approximately five times the number of approaches to adult males, compared to MLS animals, though the group difference was not significant (see lower panel of Table 1).

For two independent samples, cluster analyses revealed the existence of two types of Low-Sociable adult male rhesus monkeys. Whereas TLS and MLS animals were not significantly different in the number of social initiations directed at adult males, they differed substantially in their initiations to adult females and to juveniles/infants: MLS animals showed significantly more such initiations than did TLS animals. For an adult male rhesus monkey, adult females, juveniles, and infants might be considered relatively “safe” targets of social opportunity. Adult males mate with adult females (although our observations were not conducted during the breeding season), and sometimes play with juveniles and infants. In contrast, adult male interactions are more physically risky, and are frequently characterized as competitive and aggressive [53]. One might expect that a higher degree of social motivation and/or skill would be required to overcome such a risk; in fact, while MLS and TLS animals did not differ in their frequencies of approach or walkby to adult males for either Sample (all *p*>.10), HS monkeys did show significantly higher levels of approach to adult males (S1: Welch's *F*(1,41.64)  = 9.95, *p*<.01, est. ω^2^ = .40; S2: Welch's *F*(1,52.44) = 5.08, *p*<.05, est. ω^2^ = .06) than did animals in the combined LS group.

While we believe our cluster procedure identified two subsets of LS animals, it is possible that our identification of MLS animals as low in Sociability (reflecting the individual traits of warm, affiliative, and *not* solitary) might simply reflect an error; perhaps these animals really were high in Sociability after all, but were mischaracterized during the field cage observations. If this were the case, then MLS animals should be more similar to HS animals not only for tentative social initiations (approaches and walkbys), but for more complex social interaction as well (proximity, contact, and grooming). If MLS animals were characterized correctly, however, we would expect similarity to HS animals for tentative behaviors, but less similarity for more complex behaviors.

**Figure 2:**
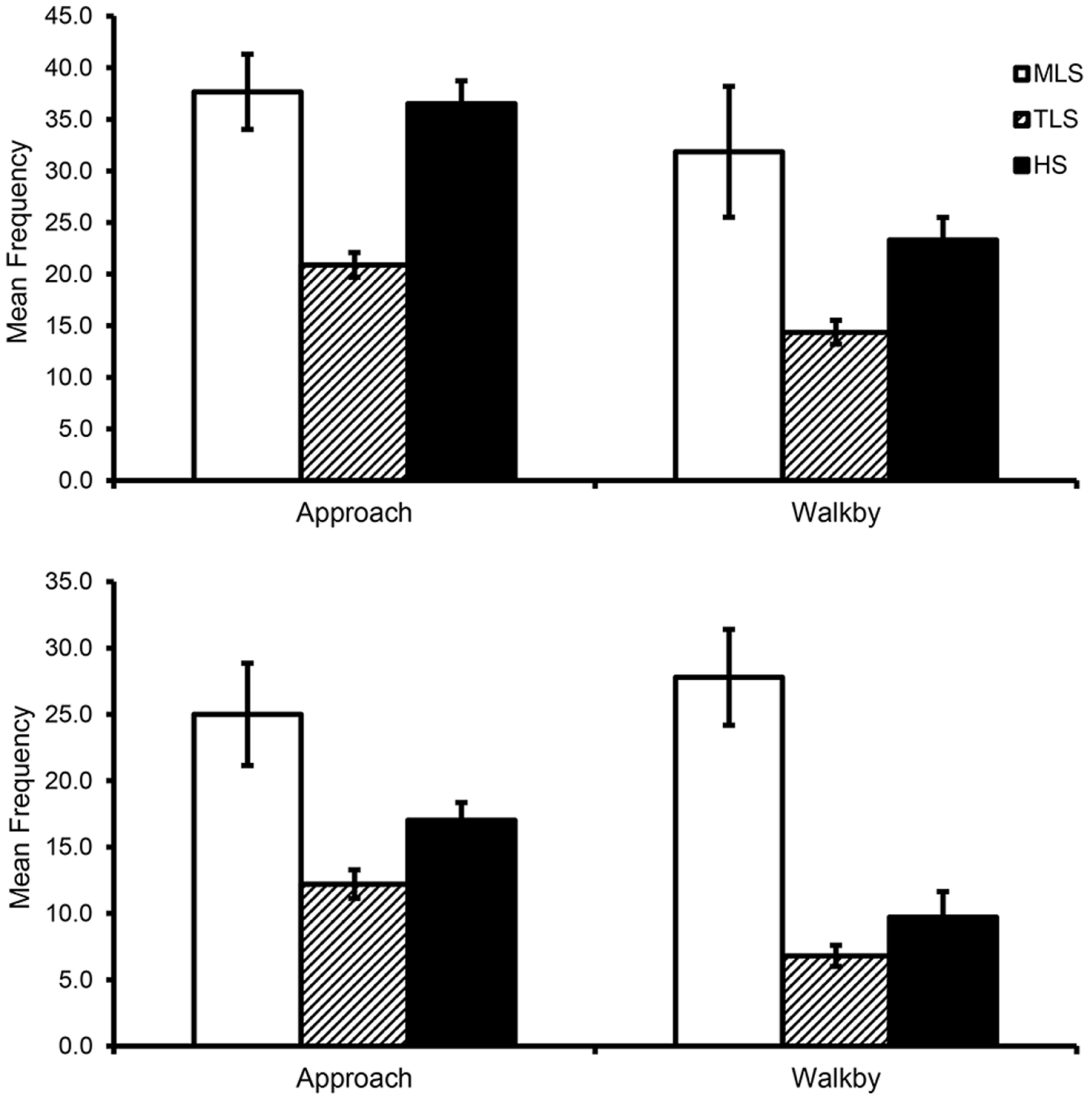
Differences between three groups of monkeys, from two samples, on measures of tentative social interaction. A. Manifestly low sociable (MLS) and high sociable (HS) monkeys from Sample 1 show comparable levels of approach and walkby, and frequencies for both groups are significantly higher than for those of the truly low sociable (TLS) group. B. MLS and HS monkeys from Sample 2 show comparable levels of approach to each other, but are significantly higher than those among TLS monkeys.

To address the alternative explanation of mischaracterization, we first compared MLS, TLS, and HS animals on the total frequencies of approach and walkby (regardless of target). Significant group differences (using Welch's test) were evident for Sample 1 (approach: *F*(2,11.19)  = 23.94, *p*<.001, est. ω^2^ = .43; walkby: *F*(2,10.19)  = 8.94, *p*<.01, est. ω^2^ = .21): for both behaviors, Bonferroni-corrected t-tests showed that mean values were significantly greater for MLS and HS animals compared to TLS animals (Figure 2A); MLS and HS monkeys did not differ from each other. Group differences were also found for Sample 2 (approach: *F*(2,65)  = 7.48, *p*<.01, η_p_^2^ = 0.19; walkby: *F*(2,65)  = 10.25, *p*<.001, η_p_^2^ = 0.24): for approach, MLS and HS animals (which did not differ from each other) had significantly higher frequencies compared to TLS animals, and for walkby, MLS animals had significantly higher frequencies than both TLS and HS animals (Figure 2B). These results are generally consistent with the idea that MLS animals show levels of *tentative* social initiation that are generally similar to those shown by HS animals.

Next, we contrasted the three groups of animals on total frequencies of more complex social interaction: proximity (remaining within arm's reach for more than 3 sec), contact (physical contact), and grooming initiated (picking through fur). Significant group effects were found for proximity, contact, and groom-initiate for both Samples (S1: *F*(2,57)  = 34.51, *p*<.001, η_p_^2^ = 0.55; *F*(2,57)  = 27.25, *p*<.001, η_p_^2^ = 0.49; *F*(2,57)  = 7.07, *p*<.01, η_p_^2^ = 0.20, respectively; S2: *F*(2,65)  = 9.75, *p*<.001, η_p_^2^ = 0.23; *F*(2,65)  = 3.87, *p*<.05, η_p_^2^ = 0.11; *F*(2,65)  = 3.18, *p*<.05, η_p_^2^ = 0.09). Bonferroni-corrected post-hoc tests for Sample 1 showed that for proximity, contact, and groom-initiate, frequencies for HS animals were significantly greater than for MLS or TLS animals, which did not differ. For Sample 2, HS animals had significantly higher frequencies than TLS animals for all three behaviors as well; mean values for MLS animals were nonsignificant and intermediate for proximity and contact, but were lowest of the three groups for groom-initiate.

Together, these data are consistent with the idea that two classes of low-Sociable animals exist among adult male rhesus monkeys. Members of one class generally show low social output, and we have characterized them as “Truly Low-Sociable.” Members of the second class of animals show levels of social interest that are more complicated, and are referred to as “Manifestly Low-Sociable”. On the one hand, MLS animals appear interested in social interaction, inasmuch as their frequencies for behaviors that might start interactions – approaches and walkbys – were comparable to those displayed by HS animals. On the other hand, they seem unable to convert those initial attempts into the kinds of social interaction that are commonly found among this species: remaining within arm's reach of another (proximity), sitting in contact, or grooming. For these behaviors, their frequencies were significantly lower than those of HS animals (S1), and not significantly different from TLS animals (both Samples). Further understanding of the psychological underpinnings of these two forms of Low-Sociability prompted our studies reported in the next section, which were performed with members of Sample 1.

## Study 3 – Experimental Social Probes

To better understand the motivational differences between MLS and TLS animals, we examined responses to three types of experimentally manipulated social stimuli involving 1) videotaped displays of aggression, fearfulness/submissiveness, or nonsocial/neutral behavior by unfamiliar adult males (***Video Playback***), 2) a human displaying low vs. high challenge behavior (***Human Intruder Challenge***), and, 3) brief group formations of previously unfamiliar animals (***Social Groups***). Because we believe MLS animals have greater social interest compared to TLS animals, we expected that MLS monkeys would be more responsive than TLS monkeys during conditions that present the animals with opportunities for affiliation (the fearful/submissive video, and the low-challenge intruder trials; see below). Moreover when forced into a social situation with other adult males, we expected the MLS animals to make more tentative social overtures (approach, walkby) than would TLS animals, whom we expected would show greater evidence of anxiety, inasmuch as their choice to avoid other animals was low.

### Methods

#### Subjects and housing

Following the field cage assessments, 18 LS animals (5 MLS, 13 TLS) from Sample 1 were relocated to indoor housing in standard-sized (0.8×0.8×1.0 m), individual cages. Animals were fed twice daily, water was available ad lib, and rooms were on a 12∶12 LD cycle. Animals received foraging enrichment daily, received in-cage object enrichment (e.g., kong toys, coconuts), and were given video enrichment on a regular basis. Probe tests began six months after relocation; animals were an average of 8.2 years, 8.8 years, and 9.3 years of age at the start of the Video Playback, Human Intruder, and Social Groups probes, respectively. Animals were a mean weight of 11.9 kg (range: 9.0 to 13.9 kg), and were healthy throughout testing. All behavioral observers were blind to Sociability status of the animals, and for each test, observers demonstrated better than 85% agreement on behavior coding. Data for all studies are presented as Supporting Information in file Data S1.

#### Video playback

Procedures were identical to those of a previous study, and used the same equipment and stimuli [54]. Briefly, animals were transported to a test room and placed in a viewing cage, where they were exposed daily to a 10-min color videotape. Three videotapes were used, in counterbalanced order, and animals were exposed to each for five consecutive days. Each videotape depicted a different unfamiliar adult male rhesus monkey displaying either aggressive (threats, lunges, toothgrinds), fearful/submissive (grimaces, lipsmacks, withdrawal), or nonsocial (visual, tactile, and oral exploration) behaviors (see [55] for definitions of the behaviors). A video camera located above the display monitor recorded the responses of the viewing monkeys, and videotapes were later coded by observers using The Observer software package [56]. Means were computed across the five days for each tape, data were transformed as needed to satisfy the assumptions of the test, and differences between MLS and TLS animals were analyzed by a factorial ANOVA treating video as a within-subjects variable. Our expectation was that, owing to their greater social interest, MLS animals would be more likely to show behavioral differences based on the content of the videotapes, compared to TLS animals. The behavioral domains examined included viewing behavior, activity, affiliative behaviors, and agonistic behaviors. Table 2A indicates (and defines) the specific measures in each domain.

**Table 2:** Behavioral definitions for Video Playback and Human Intruder probes.

A. Video Playback	Viewing behavior	Gaze aversion: duration looking away from the video monitor at more than a 45 degree angle.
	Activity	Position changes: frequency of moves from front to back of cage (and vice versa) minus 1 (for the starting position).
	Affiliation	Lispmack: Rapid lip movement usually with pursed lips and accompanied by a rhythmic smacking sound.
		Grunt vocalization: deep, soft, muffled, low intensity vocalization, which is almost a gurgling sound.
	Agonism	Fear grimace: Exaggerated grin with teeth showing.
		Threat: facial expression comprising some combination of ear flaps, lunges, open mouth stare, bark vocalizations, and/or head bobbing.

B. Human Intruder	Position	Front of cage: animal's head is in front half of cage (near intruder).
	Affiliation	Lipsmack: Rapid lip movement usually with pursed lips and accompanied by a rhythmic smacking sound.
	Agonism	Fear grimace: Exaggerated grin with teeth showing.
		Threat: facial expression comprising some combination of ear flaps, lunges, open mouth stare, bark vocalizations, and/or head bobbing.
		Cage shake: Grasping of cage parts and shaking.

#### Human intruder

On each of five consecutive days, each animal's behavior was recorded in its home cage during a Human Intruder Challenge [44], which comprised four consecutive 30-sec. trials per day. In two low-challenge trials, an unfamiliar human laboratory technician presented her left profile from 1.0 m and from 0.5 m distance. In two high-challenge trials, the technician maintained direct eye contact (i.e., stared at the monkey) from the far and near positions. A second technician recorded affiliative (e.g., threat, lipsmack) and positional behaviors of the subject every five seconds during each trial. Means were computed across the five days of testing; ANOVA (or the Mann-Whitney test) was used to contrast groups, with challenge condition as a within-subjects variable in ANOVAs. Because these animals were well-adapted to indoor housing and human presence [57], we hypothesized that MLS animals would show greater affiliation toward the human during the low-challenge trials (as indicated by affiliative responses) compared to TLS animals, but greater responsiveness (as indicated by activity and agonistic responses) during the high-challenge trials. Table 2B shows definitions of behaviors assessed in each domain.

#### Social groups

The 18 LS animals were randomly assigned to Stable or Unstable social conditions (see [47]). Animals in the Stable condition met for 100 min daily in the same 3-member groups. In the Unstable social condition, animals met for an equivalent time in groups of varying size that changed daily (two-, three-, and four-member groups were formed each day from among the pool of 9 animals). All groups met in cages constructed of chain link and measuring 1.8 m×3.1 m×2.2 m. Frequencies of social and emotional behaviors were recorded each day during four 5-min. sessions using “all occurrences” sampling [50] and durations of social states (proximity, contact, and groom) were recorded in separate 5-min daily sessions using focal animal sampling [50]. Data were averaged over the seven days of observation. Our measures of interest were frequencies of approach and walkby, and durations of proximity, contact, and groom (definitions are identical to those used in Study 2, above), with the aim of determining whether MLS and TLS animals maintained the group differences seen in Study 2, despite very different physical and social conditions. Because there were limited opportunities for animals to escape each others' initiations (unlike in the half-acre enclosures in Study 2), we also examined scratch and yawn, two behaviors indicative of anxiety or tension, and predicted that TLS animals would show more of these behaviors. Preliminary analyses showed no MLS-TLS group differences in behaviors based on social condition (all p>.25), consequently data from both Stable and Unstable groups were combined for a pooled ANOVA contrasting MLS and TLS animals. Twelve of the 18 animals had been inoculated with simian immunodeficiency virus on the day before the first day's observations, and six served as saline controls; analyses revealed no significant effects of inoculation condition (all p>.12; a common finding at this early stage of infection [58], [59]), so SIV and control groups were also combined.

### Results and Discussion

#### Video playback

**Figure 3:**
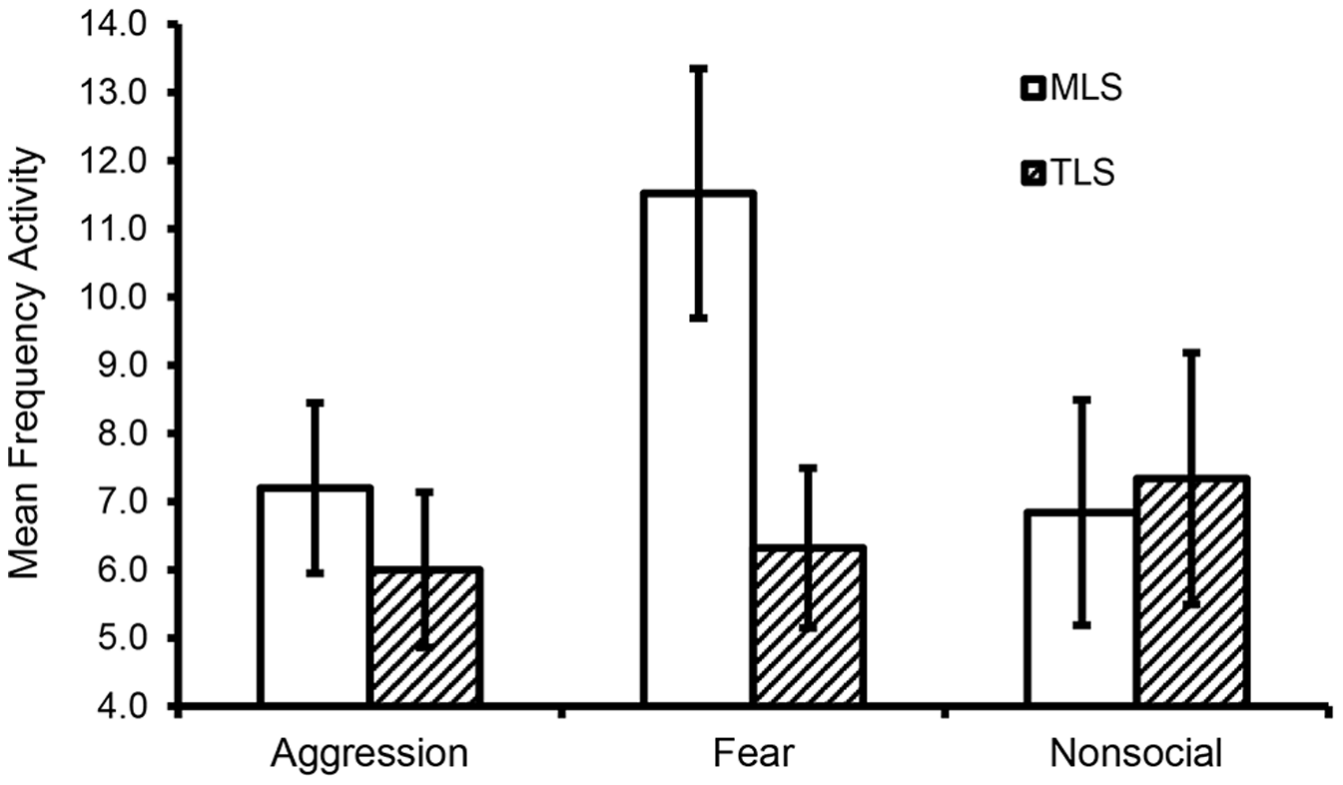
Group differences in activity during Video Playback probe. Manifestly low sociable (MLS) monkeys display greater activity (position changes between front and back of cage) than do truly low sociable (TLS) monkeys while watching the fearful/submissive videotape. No group differences were found for the Aggression or Neutral videotape displays.

As expected, MLS animals showed greater behavioral responsiveness to the videotaped stimuli based upon the content of the tape. The fearful/submissive tape, in particular, was most effective in altering the behavior of MLS animals, while the responses of the TLS animals were consistent across the three tapes. This was indicated by results for the interaction of stimulus tape (neutral/fear/aggression) × group (MLS/TLS): MLS animals a) moved more frequently between the front and back halves of the cage (*F*(2,32)  = 4.54, *p*<.05, η_p_^2^ = 0.22; Figure 3) only during the fearful/submissive tape; b) had higher durations of gaze aversion compared to TLS animals during this tape (*F*(2,32)  = 3.09, *p* = .059, η_p_^2^ = 0.16) (MLS mean (SE)  = 377.1 (77.3) sec vs. TLS mean (SE)  = 328.9 (27.4) sec) while durations for the aggressive and nonsocial tapes were comparable for TLS and MLS animals (ranging from 323.5 to 342.7 sec); and c) displayed affiliative grunt vocalizations only during the fearful/submissive tape (F(2,32)  = 3.445, p<.05, η_p_^2^ = 0.18), whereas TLS animals displayed levels that were comparable across the three tapes and were only 1/6 to 1/8 as frequent. There were no differences between groups in levels of agonistic behavior, or for other measures described in Table 2. Together, these results indicate that the MLS animals responded differently based on the type of behavior seen on the stimulus tapes while the TLS animals did not, and the display of more activity, affiliative vocalizations, and active management of gaze (an important skill when attempting to engage other adult males) suggests the MLS animals were making more active attempts at affiliating with the socially safe and/or potentially subordinate stimuli – an inference consistent with their presumed greater social interest.

#### Human intruder

**Figure 4:**
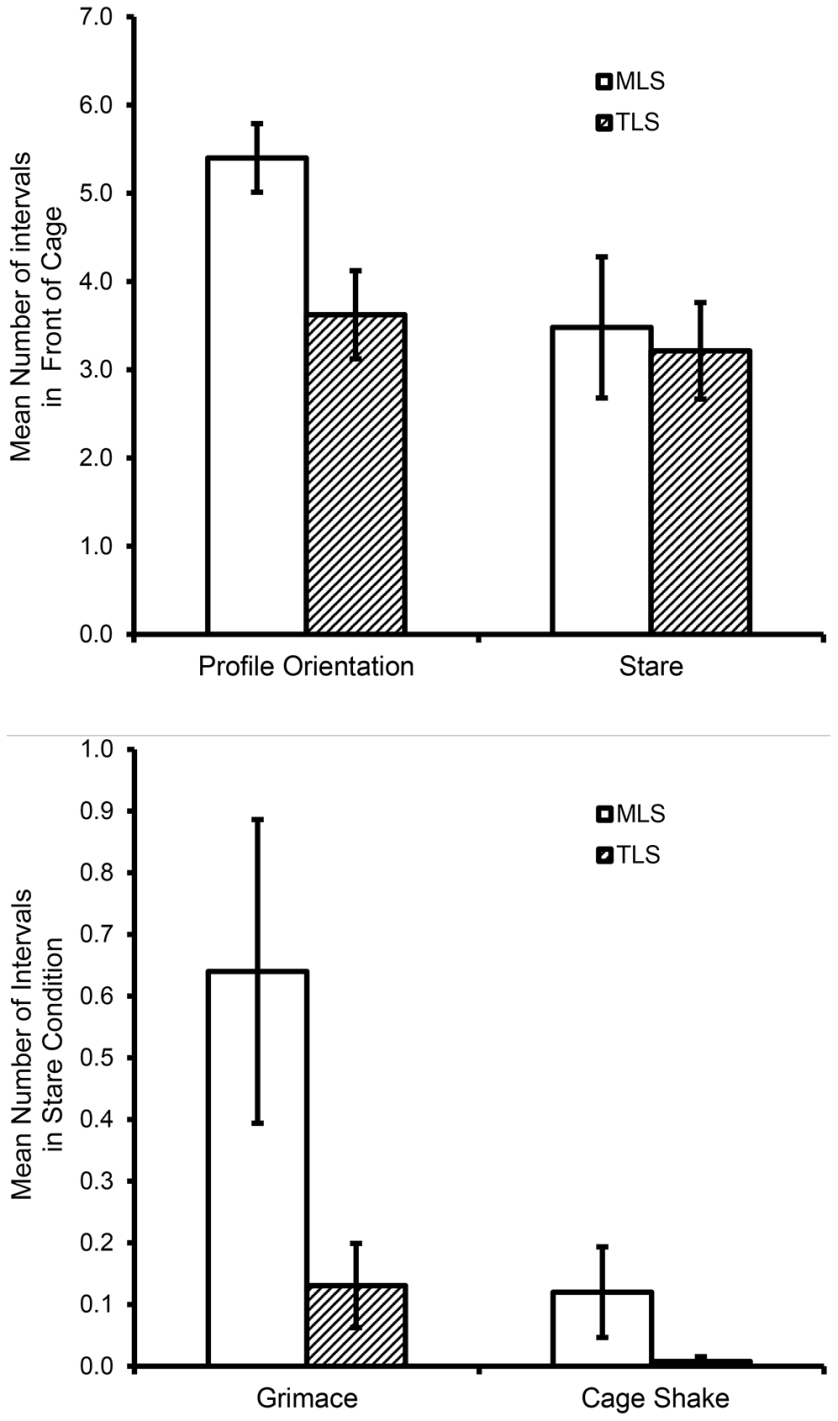
Group differences in positional and agonistic responses during a Human Intruder challenge. Manifestly low sociable (MLS) monkeys A) are more frequently in the front of the cage than are TLS monkeys, but only in low-challenge (profile orientation) conditions, and B) show higher frequencies of grimace and cage shake during high-challenge (stare orientation) conditions.

MLS animals were more socially responsive to the presence of an unfamiliar human than were TLS animals, and generally differentiated more clearly between the low- (profile) and high-challenge (stare) conditions. Frequencies of proximity to the human (i.e., location in the front of the cag) were significantly higher among MLS animals in the profile condition compared to the stare condition, while frequencies for the TLS animals were not different (indicated by a significant interaction (*F*(1,16)  = 6.60, *p*<.05, η_p_^2^ = 0.29; Figure 4A). MLS animals showed more agonistic responding as well; all occurrences of grimace, cage shake, and threat were seen only in the challenge condition, and MLS animals showed higher levels of grimace and cage shake (grimace: Mann-Whitney U = 12.0, *p*<.05, effect size (r)  = .52; cage shake: Mann-Whitney U = 15.0, *p*<.05, r = .56; Figure 4B) compared to TLS animals. Grimace and cage-shake are generally considered to reflect fear and aggression, respectively, and the MLS animals displayed nearly all recorded instances of each. As with the Video Playback probe, these results are also consistent with the idea of greater responsiveness to social stimuli among MLS, compared to TLS, animals, and greater differentiation among social conditions.

#### Social groups

**Figure 5:**
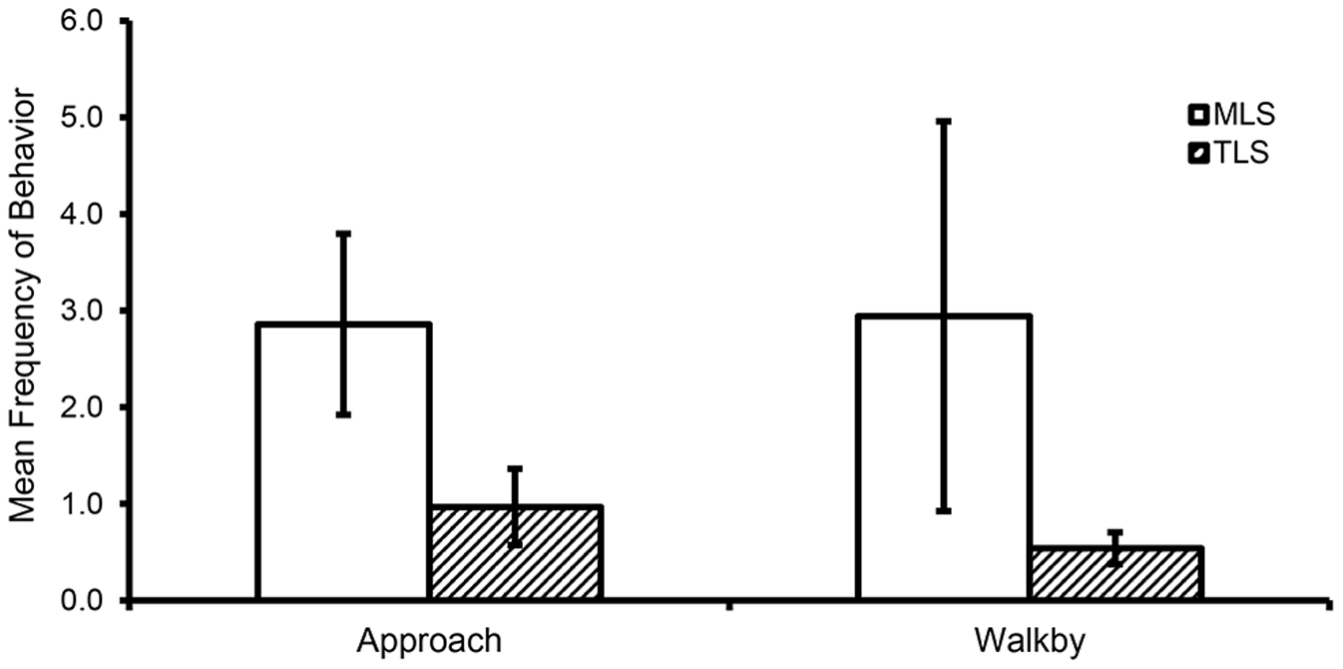
Group differences in behavioral measures of tentative interaction during the Social Groups probe. During social interaction with other adult males, manifestly low sociable (MLS) monkeys show higher frequencies of approaches and walk-bys compared to truly low sociable (TLS) monkeys.

As in Study 2, MLS animals showed significantly higher frequencies of approach and walkby than did TLS animals (Figure 5) (approach, *F*(1,16)  = 4.94, *p*<.05, η_p_^2^ = 0.24; walkby [log-transformed for analysis]: *F*(1,16)  = 5.61, *p*<.05, η_p_^2^ = 0.26, respectively). To examine the consistency of this behavior across the seven days of testing, we examined the number of days that each MLS and TLS animal showed these behaviors, and found that MLS animals displayed approach and walk-by on more days than did TLS animals (approach means (SE): MLS = 5.8 (0.73) days, TLS  = 2.3 (0.74) days, *F*(1,16)  = 7.37, *p*<.05, η_p_^2^ = 0.32; walk-by means (SE): MLS = 4.6 (0.81) days, TLS = 2.0 (0.52) days, *F*(1,16)  = 7.08, *p*<.05, η_p_^2^ = 0.31). As expected, groups differed in frequency of yawn (but not scratch), with higher frequencies recorded for TLS than for MLS animals (MLS mean (SE)  = 2.3 (0.57) yawns, TLS mean (SE)  = 4.5 (0.52) yawns, *F*(1,16)  = 5.77, *p*<.05, η_p_^2^ = 0.27). Finally, we found that MLS animals had significantly higher durations of proximity across the seven days, compared to TLS animals (MLS mean (SE)  = 105.1 (40.2) sec; TLS mean (SE)  = 32.7 (14.7) sec; F(1,16)  = 4.57, p<.05, η_p_^2^ = 0.22). These results suggest that, approximately 1.5 years after the observations in Study 2 concluded, animals identified as MLS continued to show greater social interest compared to TLS animals as evidenced by greater display of approaches and walkbys (and higher durations of proximity, but not contact or grooming), despite the very different testing (a relatively small, indoor cage) and social (groups composed of only 2–4 adult males) conditions. Thus, even in the absence of “safe” partners (e.g., juveniles, females), MLS adult males will indeed attempt interaction with other adult males, and will do so with greater persistence than do relatively unmotivated TLS monkeys, who respond with anxious behavior.

## General Discussion

There is growing evidence that people are at greater risk for poor physical and psychological health outcomes when they perceive their existing social relationships as inadequate relative to their preferred level of social involvement (see above). While we have some information on the biological mechanisms underlying the relationship between loneliness and health [60], study of such mechanisms could be greatly enhanced by development of an animal model of loneliness, which could enable experimental and mechanistic studies of biological processes that could mediate this relationship. In the present report, we propose a nonhuman primate model of loneliness.

While the idea of a “lonely” animal may seem anthropomorphic, from an evolutionary perspective there is no reason why some individuals cannot have a mismatch between social interest and social attainment. For any social species, the conspecific environment has been an important factor in shaping the behavior and biology of individuals, and individuals have evolved mechanisms to enable them to reap the benefits of sociality while minimizing the costs; in a very real sense, a species' sociality is embedded in its basic biology [61], [62]. Variation exists in social tendencies, however, even within species [63], and this variation may reflect fitness advantages during critical periods of a species evolutionary history; for example, when a pathogen was epidemic, individuals with reduced affiliative tendencies may have had a fitness advantage [38], [64], [65]. In more benign times, low social interest may carry few costs, and as described above, introversion in humans, reflecting a preference for low levels of affiliation, often is not associated with poor health outcomes. But what conditions might encourage a mismatch between social interest and social attainment? Our human study (Study 1) suggests an important concept is social choice, the extent to which one's social activities are under one's control. In that study, we found that individuals with low social choice and with small social networks showed the most loneliness. Can the concept of social choice operate in nonhumans?

In multimale, multifemale groups of nonhuman primates, dominance hierarchies often affect choice, by defining which animals have priority of access to particular resources – food, mates, grooming partners [66]. Even within hierarchically structured societies, however, dominance does not explain all of the variance in social choices; among juvenile rhesus monkeys, for example, sex, similarity in temperament, and kinship predict which animals maintain stable friendships, above and beyond social rank [67]. In the present study, we found that social choice was manifested in frequencies of interaction with members of various age/sex classes of group-mates. Two groups of animals were identified (Study 2) that had been judged by experienced behavioral observers as showing high or low levels of social interest, and these subjective judgments were confirmed using objective measures. Cluster analyses of behavioral data collected from Low-Sociable animals, from two independent samples, revealed two types of low sociability: TLS animals, whose social choices reflected low levels of interaction across age/sex categories, and MLS animals that showed more initiations to “safe” targets which, for adult male rhesus monkeys, include immature animals and adult females. The distinction between TLS and MLS animals was not related to differences in age, rank, or Sociability in either sample. When compared to High-Sociable animals, MLS animals in both samples showed levels of simple social initiations (approaches, walkbys) that were comparable, but levels of complex social interaction that were either significantly lower than levels shown by HS animals or were intermediate between HS and TLS animals. In the animals' familiar cages, then, the MLS animals displayed high social interest, as indexed by high levels of social initiation overall, but seemed unable to “convert” these initiations into more complex interaction. Finally, in Study 3, in which MLS and TLS animals were placed in a variety of artificially constructed social situations, the greater social interest of the MLS animals was most evident – they were more responsive to videotapes of monkeys displaying fearful/submissive signals (but not aggressive signals), more affiliative toward an unfamiliar human under benign (but not challenging) conditions, and when placed into small social groups with only other adult males, again displayed more approaches and walkbys than did TLS monkeys.

While we believe the MLS animals might constitute a useful nonhuman primate model of loneliness, we acknowledge several limitations of our study. First, we recognize that the correspondence between the human and monkey data may be imperfect. While we believe that our HS and TLS animals likely correspond to the high choice/large network and high choice/low network groups, respectively, from our human study, and that MLS animals most closely fit the low choice/small network pattern (at least based on complex social interaction), the monkey study does not have a parallel to the “low choice, large network” group. In the human study, members of this group were not significantly different from those in the high choice/small network group (which we consider similar to TLS monkeys), but we do not consider that the concept of “low choice, large network” is consistent with our characterization of “truly low sociable.” More work is needed to determine whether such a grouping exists for adult males. We suspect, however, that low choice and a large network may be more evident among adult female monkeys, who remain in the social group in which they were born for their entire lives, surrounded by kin; kinship may provide females with a large social network, but kinship can also constrain choices. Our use of males in the monkey studies might well contribute to the imperfect fit between the monkey and human data.

Second, we note that our sample sizes for the MLS subgroup were small; in each sample, only 5 animals (16% of LS animals) were found that fit the pattern of high levels of social initiations but low levels of complex social interaction. Our replication of these results with a second, independent sample, however, provides more assurance of the existence of a small set of animals that show a mismatch between social interest and social attainment. Nevertheless, given the resources needed to identify TLS animals, a higher-throughput approach to identification would make this model most valuable.

Third, while we interpret the variation in our MLS animals' social initiations as reflecting social choice, we acknowledge that we do not fully understand the psychological underpinnings of such choice. Do MLS animals choose their preferred targets out of heightened sensitivity to social threats and rejection, as is seen in humans [10]? Or are MLS animals deficient in some way in their social behavior (e.g., [54])? Are MLS animals perceived as unattractive partners by others? These questions remain to be answered.

Finally, while we believe our behavioral data suggest a possible nonhuman primate model of loneliness, confidence would be strengthened by finding physiological measures in MLS monkeys that parallel those found in lonely humans (e.g., [62]). Should such parallels be found, then the more rapid development of monkeys combined with their greater accessibility for experimental manipulation, tissue sampling, and pharmacological treatment could be of great value in clarifying developmental contributors to loneliness, as well as the behavioral and physiological mechanisms by which loneliness influences individual well-being, health, and fitness [3], [6]–[8].

## Supporting Information

Checklist S1 **ARRIVE checklist.**
(PDF)

Data S1 **All data for the studies in the present report.**
(XLSX)
